# Book Review

**Published:** 1924-01

**Authors:** 


### Reviews of Books.

**Angina Pectoris.** By Sir James Mackenzie, M.D., F.R.S. Pp. xvi., 253. London: Oxford Medical Publications. 1923. Price 30s. net.—Any book written by Sir James Mackenzie is interesting to read, and his latest work on *Angina Pectoris* is no exception to the rule. He has published a lot of it before, but it was necessary to repeat it in order to carry the argument through to the end in its proper sequence. He has brought physiology in to explain the symptoms of angina, and has given a much stronger explanation of the disease than has been done in the past. Chapter xv. is especially interesting, in which are described the changes that take place with advancing years, and it is shown that many individuals who, from post-mortem appearances, should have suffered from angina were spared, because degeneration of arteries in other parts had limited their power of effort so much that no extra work could be put upon the heart. There are chapters given up to the description of the mechanism of secondary angina and its differentiation from primary angina. The term "secondary," as described in the text, is peculiarly apt, since it leads the physician to look further for the primary cause. It is not to be found in the heart. At the end of the book is an appendix containing a brief description of 160 cases and post-mortem records of twenty-two cases, from which one can see the reasons for the deductions given in the text. It is a book well worth reading, and greatly advances our understanding of the disease.

**Elements of Surgical Diagnosis.** By Sir Alfred Pearce Gould. Sixth Edition, revised by Eric Pearce Gould, M.D., M.Ch. Pp. xiv., 739. London: Cassell and Company Limited. 1923. Price 12s. 6d. net.—When a book reaches its sixth edition and its fortieth year there is not likely to be much that will make a review of it striking. But the mere historical fact is a sufficient evidence of public esteem. However, eloquent tribute is paid to the rising importance of roentgenology in Surgery by the number of skiagraphic illustrations being doubled in the 1923 compared with the 1914 edition, and by the much-needed warning that the interpretation of plates "is not the extremely easy matter that some have thought." Those of us who learn best from the written word can want no more reliable and comprehensive guide to surgical diagnosis whether in the abdomen or out of it. The book is characteristically English, and not the least satisfactory feature of it is the opening chapter on the method of diagnosis. Here are clearly defined axioms, the mastery of which means that the student's foundations of diagnosis are well and truly laid.

**Hygiene and Public Health.** By LOUIS C. PARKES, M.D., D.P.H., and HENRY R. KENWOOD, C.M.G., M.B., D.P.H., Seventh Edition. Pp. xi., 783. London: H. K. Lewis & Co. Ltd. 1923. Price 20s. net.—It is generally safe to say that any book which has reached its eighth edition is one which has successfully met a definite need, and this is undoubtedly true of the volume before us. There are few practitioners or students of Public Health indeed who are not as familiar with the appearance, as they would like to be with the contents, of their "Parkes and Kenwood," which is as nearly indispensable to them as any book could be, since it contains most of the information they are likely to need and little they can afford to ignore. In an edition which is stated to have been thoroughly revised and enlarged one naturally looks to see what is said about modern developments. One is seldom disappointed. The sections dealing with Maternity and Child Welfare, Venereal Disease, Milk (including references to Graded Milk), and so forth, are all based on the most recent enactments, while controversial matters are described fairly and without bias. The book is endowed with a power of growth which shows it to be a live thing, and ensures a continuance of that career of usefulness and success which it has hitherto achieved in so signal a manner. There are one or two suggestions we would venture to make for the next edition. Would it not be better to eliminate all references to those numerous Acts which are repealed by the Education Act, 1921 (*e.g.*, see page 636), and to mention the consolidated Act only? We doubt whether the maternity benefits under the National Insurance Acts are exactly as given on page 622. Probably some Medical Officers in charge of Infectious Disease Hospitals, from which cases of uncomplicated Scarlet Fever are sent out in five or even four weeks as a matter of routine, would question the statement that the "usual duration of infectiveness" of that disease is "from six to eight weeks" (page 452). In the admirable section on School Work some reference to the special requirements of the Board regarding Medical Inspection in Secondary Schools would be helpful, and so would a brief description of Intelligence Tests and Questions.

J. F. BLACKETT.

**A Text-Book of Pharmacology and Therapeutics.** By E. POULSSON, Professor of Pharmacology in the University of Christiania. English Edition edited by W. E. DIXON, M.A.,

M.D., F.R.S. Pp. xi., 519. London: William Heinemann Ltd. 1923. Price 25s.—At the present time two types of text-book on pharmacology are available for practitioners and students, and both have their own sphere of usefulness. In one type the majority of space is devoted to the description of experiments which have led to conclusions as to the action of drugs, and comparatively little information is given with regard to the practical application of knowledge so acquired. The other type, to which the text-book under consideration belongs, gives more attention to the practical requirements of clinicians, and we should describe Poulsson's book as one which can be read with pleasure and profit by a medical man or student at the end of a hard day's work. It is obviously impossible for a reviewer to read every book from beginning to end, but we have selected a number of important drugs, and find that the sections dealing with their action are written in a pleasing and accurate manner; for example, there is an excellent chapter on alcohol in which the views of various workers are impartially discussed. The references to the use of camphor illustrate in an admirable way that there is no reason why a drug should not be valuable in clinical medicine for diseased conditions because it apparently has little effect on normal animals. The description of drugs acting on the vegetative nervous system is also good, but we should prefer to substitute "autonomic" for "vegetative." In this connection it is hardly correct to refer to physostigmine as one of the para-sympathetic poisons, and it would be better to classify it as a para-sympathomimetic drug. As an example of the care which has been taken to render the text-book up to date, we note with pleasure a concise description of that very useful compound benzyl-benzoate. The section on antipyretic drugs is carefully and simply written, and demonstrates the importance of realising the connection between chemical constitution and pharmacological action. A slight error has crept into this section on page 201, where it is stated that febrifuges that are based upon para-amido-phenol are *more harmless* than aniline compounds. We have derived much pleasure from the book, and congratulate all who have contributed to the production of this volume on the excellence of their work, and strongly recommend it to practitioners and students.

O. C. M. Davis.

**L'Hématoblaste, troisième élément du sang.** Par Georges Hayem. Avant-propos et annotations par L. Rivet. Pp. 295. Paris: Les Presses Universitaires de France. 1923. Price 25.00 Fr.—This deceptively small-looking volume contains much of interest and more of controversial matter. It consists of two distinct portions: the first, written by the veteran hæmatologist Hayem, is a compendium of his numerous publications on the origin and functions of the blood platelets, work spread over the years 1877–1915 : the second portion is an annotation on the text of the first, with a selection from the more modern instances by L. Rivet, Hayem's disciple. Many of the theories enunciated find few supporters even in France and still fewer elsewhere. About one-third of the volume deals with Hayem's theory of hæmopoiesis, *i.e.* the blood platelets, which he admits to be the probable offspring of the megacaryocytes, are the first phase of the red-blood corpuscle. He traces the transition through the poikilocyte and the microcyte to the fully-formed erythrocyte. He regards the erythroblast of the bone marrow as of little or no importance in blood regeneration. This theory is based mainly on two observations : (1) The appearances and staining reactions of the platelet in wet and dry films, (2) the increase of platelets prior to an increase of red-blood corpuscles ; the latter was first noted by Hayem. The first method is so full of pitfalls that any conclusion might be made : the second admits other interpretations. Rivet, ignoring much work which to an unbiased mind disproves the theory, comments on Hayem's dicta and refers to numerous publications which would appear to support the thesis. His references are peculiarly one-sided : the demonstration of the transition from normoblast to erythrocyte in cultures of bone marrow (Tower and Herm, *Proc. Soc. Biol. and Medicine,* 1916, xviii. 505) ; the clear-cut serological distinction between platelet and erythrocyte (Bedson, *J. Path. and Bact.*, 1921, 24, 469) ; numerous demonstrations of nuclear remains and centrosome in the red-blood corpuscles by various methods—and much other evidence of crucial importance in the controversy receives no mention whatsoever. The work of J. H. Wright on the platelet, published in 1910, and considered of classical importance, is accorded two brief allusions, but in spite of this we are gravely informed that it is a current belief that the platelet is the extruded nucleus of the red-blood corpuscle. Numerous other points of less importance will be questioned by any reader who keeps abreast of modern work. The section on the relationship of the platelet to the humoral changes in anaphylaxis is too sketchy to be of any value. In this section Hayem's belief that asthma, amongst other conditions equally doubtful, is a stigma of degeneration, is somewhat surprising in view of the fact that certain varieties of asthma can be conveyed passively by blood transfusion. The portion dealing with the enumeration of the platelets is very full. That describing the pathology, the anæmias, the hemorrhagic diseases and therapeutics appears to have been condensed at the expense of the earlier theoretical sections : so far as they go they are good, but much modern work, *e.g.* on the importance of the vascular and other endothelium, is partially or completely ignored. The index and binding, like those of the majority of continental publications, are poor. The illustrations are indefinite and ambiguous in the main. The book is of great interest as an epitome of thirty-eight years' work. Though much can be regarded as having been disproved, much still remains as fact. Amongst other works of importance, Hayem's contributions to the study of blood coagulation and the pathology of purpura are monumental. Perhaps his lifelong insistence on the importance of the blood platelet in pathology, although we cannot accept all his opinions on their origin, is a work of still greater merit: directly or indirectly, he stimulated a research which already has led to great strides in our knowledge of an important group of diseases, which remained obscure until he commenced the study of the blood platelet.

A. T. TODD.

**Theories and Problems of Cancer.** By CHARLES EDWARD WALKER, D.Sc., M.R.C.S., L.R.C.P. Pp. 126. London: University Press of Liverpool. 1923. Price 5s. net.—The object of this handbook of 126 pages is to set out for the lay reader the nature of the problems which are occupying the minds of those engaged in cancer research, and to supply him with the salient facts known about new growth in general. The majority of lay readers will, we feel sure, have but the faintest grasp of these questions after reading the book, but for the trained medical reader who has not followed the undoubted progress of our ideas on this subject the volume will have considerable interest. The author's arguments are easy to follow and are shorn of unnecessary detail. He convinces one that, in searching for a cause of neoplastic growth, we are engaged in a problem of cyto-physiology of a fundamental character. The author's valuable work in this field with Farmer and Moore is explained in simple language, and it is pointed out how frequently their theory is misquoted in text-books. In the discussion on treatment it is stimulating to find that Norgate's work on the effect of pituitrin on inoperable cancer is favourably commented upon. We fail to find any mention of Boveri's theory based on multipolar mitosis—a conception which at least deserves critical treatment.

**Diseases of the Ear.** By WM. MILLIGAN, M.D., and WYATT WINGRAVE, M.D. Pp. 191. London: Wm. Heinemann. 1923. Price 12s. 6d.—This work under review is designed as a student's textbook, and as such presents in a clear and concise form all the essentials in the diagnosis and treatment of diseases of the ear. The high standard set by the larger work on this subject by the same authors has been maintained in the present volume. The subject-matter is clearly expressed, the illustrations are good, and the difficult decision between what to put in and what to leave out has been well made. The final chapter on formulæ and pathological methods will be found of considerable help to the practitioner. Printers' errors seem conspicuous by their absence. We suggest the omission of the illustration of a hand mirror on page 8 as being of doubtful utility, and further that the Holmes nasopharyngoscope is a more useful instrument than that of Hays, which is the one recommended. The latter gentleman, by the way, seems to have acquired a superfluous "e" on page 51. We can cordially recommend this work to the student and practitioner.

**Electric Ionization.** By A. R. Friel, M.A., M.D. Second Edition. Pp. 132. Bristol: John Wright & Sons Ltd. 1922. 8s.—Although ionization as a method of treatment has been before the profession for many years, it cannot be said that the method has become widely popular. In this, the second edition of Dr. Friel's work, full details of the necessary apparatus and its technical application are given. While ionization can be used in the treatment of a large number of conditions, it is in the treatment of chronic suppuration in the middle-ear that it seems latterly to have given the most promising results. Largely owing to the work of Dr. Friel in this country, the method is employed as a routine in some of the school clinics, with apparently favourable results. The treatment of this class of case is dealt with in considerable detail and with breadth of outlook. What is claimed, apparently with justification, is that zinc ionization in one or two applications will clear up a case of chronic suppuration in the middle-ear in which this suppuration is confined to the middle-ear and not associated with deep-seated bony changes.

**The Medical Profession in India.** By Major-General Sir Patrick Hehir, K.C.I.E., C.B., C.M.G., I.M.S. Pp. v., 139. London: Oxford Medical Publications. 1923. Price 7s. 6d.—This book gives a concise account of the growth and present position of western medicine in India—of medical education and its problems, and closes with an interesting description of the indigenous medical systems, the Ayurvedic or Hindu and the Unani or Mahomedan. The general impression gained from a perusal of this work is that in spite of much splendid service the blessings of western medicine have as yet only been brought within reach of those living in the more populous centres, whereas the rural population of India is vastly in excess of the urban. The author estimates that fully 100,000,000 of the peoples of India are at present outside the reach of western medicine—that in the north-eastern area, excepting the town of Calcutta, there is but one practitioner, native or European, to every 181,000 people. Another figure given by the author gives an impressive sense of the needs of India. The Sanitary Commissioner with the Government of Bombay estimates the infant mortality in that city at the appalling figure of 500 per thousand. With facts such as these before us, it is the more to be regretted that the I.M.S. should be so unpopular in these days with the recent graduates of our home medical schools, for no part of the world calls out more loudly for their help, and no sphere offers such magnificent scope for service of the highest order. The needs of India can, however, only be fully met by India's own sons and daughters, and the hope of the future lies in these trained in their thousands as doctors, midwives, nurses, welfare workers and sanitary inspectors. Those who feel the call of India and decide to serve there will find in this book a clear and useful presentation of many of the problems and opportunities that await them in the land of their choice.

**Aromatics and the Soul : A Study of Smells.** By Dan McKenzie, M.D. Pp. ix., 164. London : Wm. Heinemann Ltd. 1923. Price 7s. 6d. net.—Dr. Dan McKenzie writes of the sense of smell, its psychological wonders and its practical importance. He claims for the British race a special fineness of appreciation of smells, sweet and other. The arresting title and prosaic subtitle of his work prepare the reader for something out of the usual. The author gives a short sketch of the present position with regard to theories of olfaction, and some of the curious experiments on which they have been built. He skilfully avoids all semblance of a text book. "He who runs may read herein or may refrain from reading, just as he pleases ; seeing that he can never be under the compulsion of remembering a single word." It is, however, when we turn to the psychological aspect of olfaction that we get the full savour of Dr. McKenzie's charm. The queerly vivid link between memory and smell, the importance of smell in folk lore and religion, the vagueness of our knowledge and vocabulary of smells, of all these and more he reminds us. The almost complete neglect of tobacco may strike some readers with a sense of omission. A most attractive little book that everyone, medical and otherwise, will find full of delight and of profit.

